# Feeding styles and child weight status among recent immigrant mother-child dyads

**DOI:** 10.1186/1479-5868-9-62

**Published:** 2012-05-29

**Authors:** Alison Tovar, Erin Hennessy, Alex Pirie, Aviva Must, David M Gute, Raymond R Hyatt, Christina Luongo Kamins, Sheryl O Hughes, Rebecca Boulos, Sarah Sliwa, Heloisa Galvão, Christina D Economos

**Affiliations:** 1 John Hancock Research Center on Physical Activity, Nutrition, and Obesity Prevention, Gerald J. and Dorothy R. Friedman School of Nutrition Science and Policy, Tufts University, 150 Harrison Avenue, Boston, MA, 02111, USA; 2 National Cancer Institute, National Institutes of Health, Bethesda, MD, USA; 3 Immigrant Service Providers Group/Health, c/o Somerville Community Corporation, 337 Somerville Avenue, 2nd Floor, Somerville, MA, 02143, USA; 4 Department of Public Health and Community Medicine, Tufts University, 136 Harrison Avenue, Boston, MA, 02111, USA; 5 Civil and Environmental Engineering, School of Engineering, Tufts University, 200 College Avenue, Medford, MA, 02155, USA; 6 Children's Nutrition Research Center, Baylor College of Medicine, 1100 Bates, Houston, TX, 77030, USA; 7 Brazilian Women’s Group, 569 Cambridge St, Allston, Boston, MA, 02134, USA

**Keywords:** Feeding styles, Children, Obesity, Immigrants

## Abstract

**Background:**

Research has shown that parental feeding styles may influence children’s food consumption, energy intake, and ultimately, weight status. We examine this relationship, among recent immigrants to the US. Given that immigrant parents and children are at greater risk for becoming overweight/obese with increased time in the US, identification of risk factors for weight gain is critical.

**Methods:**

Baseline data was collected on 383 mother-child dyads enrolled in Live Well, a community-based, participatory, randomized controlled lifestyle intervention to prevent weight gain in recent immigrant mothers. Socio-demographic information together with heights and weights were collected for both mother and child. Acculturation, behavioral data, and responses to the Caregiver’s Feeding Styles Questionnaire (CFSQ) were also obtained from the mother.

**Results:**

The children’s average age was 6.2 ± 2.7 years, 58% male. Mothers had been in the country for an average of 6.0 ± 3.3 years, and are Brazilian (36%), Haitian (34%) and Latino (30%). Seventy-two percent of the mothers were overweight/obese, while 43% of the children were overweight/obese. Fifteen percent of mothers reported their feeding style as being high demanding/high responsive; 32% as being high demanding/low responsive; 34% as being low demanding/high responsive and 18% as being low demanding/low responsive. In bivariate analyses, feeding styles significantly differed by child BMIz-score, ethnic group, and mother’s perceived stress. In multiple linear regression, a low demanding/high responsive feeding style was found to be positively associated (ß = 0.56) with a higher child weight as compared to high demanding/high responsive, controlling for known covariates (p = 0.01).

**Conclusions:**

Most mothers report having a low demanding/high responsive feeding style, which is associated with higher child weight status in this diverse immigrant population. This finding adds to the growing literature that suggests this type of feeding style may be a risk factor for childhood obesity. Further research is needed to help understand the larger socio-cultural context and its influence on feeding dynamics among immigrant families and families of lower incomes. How parents establish a certain feeding style in their home country compared to when they move to the US “obesogenic” environment, should also be explored.

## Background

In 2009, there were approximately 37 million immigrants in the United States and it is projected that by 2050, nearly one in five Americans will be an immigrant [1]. A large number of studies have shown that overweight and obesity increase with length of stay in the US. [2-7]. Immigrants often encounter financial, linguistic, and social stressors as they acculturate to a new country, which may be associated with weight gain [8]. Although most of the literature describing this transition has focused on immigrant adults, immigrant children are also at risk for becoming obese. They face many of the same challenges as their parents when confronting the realities of a new culture and family food environment [9,10]. For instance, upon arriving in the U.S., immigrants are immediately exposed to the U.S. “obesogenic” environment. This environment is characterized by the availability of inexpensive, energy-dense foods, limited opportunities for meal-oriented food preparation and for sufficient physical activity, all of which likely contributes to weight gain [2-5,11-13]. This type of environment puts additional pressures on parents as they try to navigate the food landscape in the U.S. It is important to understand the parent–child dynamic given the critical role that parents play in determining their child’s behaviors, habits, and attitudes, and dictating their physical and social environment [14,15]. Although parenting includes several different factors, parenting styles may play an important role in how children navigate the food environment.

**Figure 1:**
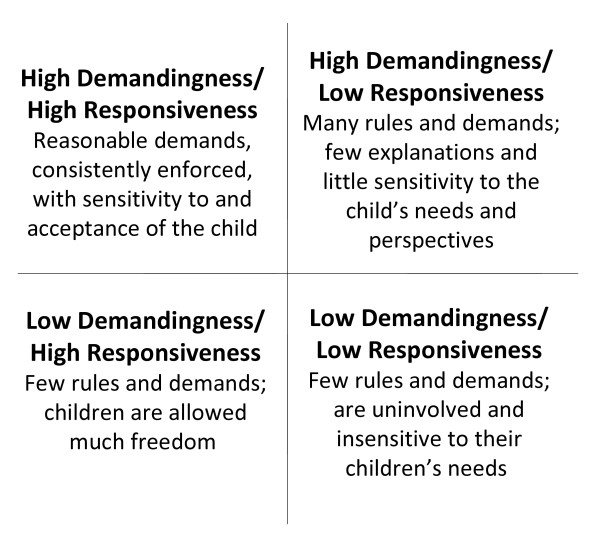
**Feeding Style Typologies [**[25].

Parenting style is a set of behaviors that reflect a certain attitude toward the child and attempts to describe the emotional climate of parent–child interactions [16]. While recent evidence suggests the influence of parenting on child overweight and obesity is complex [17], one likely pathway is the indirect influence of parenting styles on child development and behavior [18]. Parenting style may be domain specific, such that measuring a style of feeding as opposed to a style of general parenting may be more predictive of weight status in childhood because of the context-specific impact of feeding on the eating behavior of children [19]. Children’s preferences and food consumption may be shaped by feeding styles and practices [20-23], and influence energy intake [20,21,24]. The notion of feeding styles assumes that parents possess overarching styles that reflect how they interact with their children during feeding situations that can be described across two dimensions: demandingness and responsiveness. Demandingness refers to how much the parent encourages eating, whereas responsiveness refers to how parents encourage eating, that is, in a responsive or nonresponsive way. In 2005, Hughes and colleagues developed the Caregiver’s Feeding Styles Questionnaire (CFSQ) to categorize parents into four categories across those two dimensions: high demandingness/high responsiveness (i.e., reasoning, complimenting and allowing choice of appropriate foods, but also making clear expectations with regards to food consumption. Also has clear guidelines or rules that children are aware of), high demandingness/low responsiveness (i.e., using food rewards and punishments to enforce strict food rules and does not make any exceptions or adjustments based on the child’s needs), low demandingness/high responsiveness (i.e., being warm and accepting but making few demands with regards to food on the child) and low demandingness/low responsiveness (i.e., lets the child eat whatever he/she wants) [25]. This feeding typology is depicted in Figure 1.

The few studies that have explored these styles among racial/ethnic minorities found that children of parents who are responsive to their child’s emotional states but refrain from setting appropriate boundaries with their child (low demandingness/high responsiveness) are at the greatest risk for childhood obesity [19,25-28]. Given the unique situation that immigrant parents and children face in the process of acculturating to a new social, cultural and physical environment, the role of feeding styles may be particularly important in influencing child weight status. We sought to provide some of the first information about the influence of feeding styles on the weight status of recent immigrant mothers and their children. We hypothesized that children of parents with a low demanding/high responsive style would be at greater risk for overweight and obesity compared to those with a high demanding/high responsive style.

## Methods

### Study overview

The data analyzed for this study were collected at baseline (2009–2011) from Live Well, a community-based, participatory research project, which features a randomized controlled lifestyle intervention. The central premise of Live Well is that an appropriately timed intervention, co-created by community partners and academic researchers, can prevent excess weight gain in recently arrived immigrants. A total of 383 mother-child dyads had data collected at baseline. Dyads were eligible if the mother met the following criteria: resided for <10 years in the U.S., of Haitian, Latino or Brazilian descent, 20–55 years of age, not pregnant (or >6 months postpartum), has a child between 3 and 12 years of age, lives in the Greater Boston area, and willingness to be randomized. There was no weight criterion to participate in the study. Informed consent was obtained on all participants, assent for children over 7 years of age and written consent from a caregiver for children less than 7 years. Mother-child dyads attended a measurement day at a nearby community setting to complete baseline measurements. Nine child participants were found to be younger than 3 years after they were randomized: four children were aged 2.8-3.0 years, one child was 2.1 years and two were less than 2. The study was approved by the Institutional Review Board of Tufts University.

### Measurements

#### Caregiver’s feeding styles questionnaire (CFSQ)

The CFSQ is a self-administered, 31-item instrument that collects information on parenting approaches in the context of feeding (e.g., feeding styles) [25]. Each of the constructs depicted in Figure 1 is measured through a series of questions, and scored on a 5-point Likert scale (never, rarely, sometimes, most of the time, always). The feeding style dimensions are derived from parent-centered and child-centered scales. Child-centered feeding is characterized by directives that promote internalization of parental values (i.e., reasoning, complimenting); whereas parent-centered feeding is characterized by directives that promote control over children’s eating through external means (i.e., demands, rewards). Information about the development, reliability, and validity testing of this instrument is published elsewhere [25,29].

We evaluated test-retest reliability with a sub-sample of 72 participants who were enrolled in the Live Well study. The CFSQ was mailed to participants within one week of initial completion; they were asked to complete it a second time and return it within 2–3 weeks. Test-retest correlations were similar to what Hughes has reported [25,29] (r = 0.79 (p < 0.0001) for demandingness and r = 0.73 for responsiveness (p < 0.001)).

The CFSQ was translated and back-translated from English into Haitian-Creole and into Portuguese. A Spanish version of the CFSQ was available from prior work conducted by Hughes and colleagues [25,29]. The back translation was compared to the original English version to ensure the concepts were the same. Two cognitive interviews were each conducted with native Portuguese- and Haitian-Creole-speaking immigrant mothers to verify understanding of the translated CFSQ. Discrepancies in content, language, and meaning were discussed with study personnel, and survey language was modified as needed.

Reflecting a community-based, participatory research platform, all aspects of the research methodology and approach have been discussed with community partners. Live Well’s partnering organizations include the Brazilian Women’s Group, the Community Action Agency of Somerville, The Welcome Project, and the Immigrant Service Providers Group/Health and the Haitian Coalition. These organizations collectively offered the Live Well project access and knowledge of the immigrant populations involved in this research. The community partners, as well as some of the academics, believed that the use of the standard or more commonly used feeding style labels (authoritative, authoritarian, indulgent and uninvolved) had the potential to lead to erroneous ethnic-specific interpretations. Discussion centered around the labels as opposed to the inherent meaning of the constructs or the specific questions on the CFSQ. The project decided to move forward describing the feeding styles along two dimensions (demandingness and responsiveness) as they may be less subject to potential misinterpretation.

#### Body mass index (BMI)

Height and weight were obtained from mothers and children. Measurements were taken in triplicate following standardized procedures [30]. Height was measured, without shoes, to the nearest eighth of an inch using a portable Seca 214 stadiometer. Weight was measured in light clothing, without shoes, to the nearest 0.1 lb on a portable digital scale (Befour PS-6600 Portable Scale; Befour Inc., Saukville, WI). Body mass index (BMI) was calculated from the average of the three body weight and height measurements for each dyad (kg/m^2^). Each child’s BMI was transformed into a z-score relative to the age- and sex-specific CDC reference [31]. The percentile score corresponding to the z-score, and the following terminology were utilized to classify child weight categories: underweight (<5^th^ percentile), normal weight (5^th^ – 84^th^ percentile), overweight (85^th^ – 94^th^ percentile), and obese (≥95^th^ percentile) [32]. Maternal weight status was classified based on her BMI; categories: underweight (<18.5), normal weight (18.5–24.9), overweight (25.0–29.9), and obese (≥30.0) [33].

#### Covariates

Information on potential covariates was collected through the self-administered survey completed at baseline measurement. Mothers reported their child’s date of birth and gender; as well as their own age, race/ethnicity, marital status, education level, and household size. Other covariates of interest included: depression, as measured by the Center for Epidemiologic Studies Depression Scale (CES-D) [34]; psychosocial stress, as measured by the Perceived Stress Scale (PSS) [35]; country of birth; and number of years and/or months the respondent has lived in the U.S. To assess acculturation, mothers were asked: “When you think about your daily life now, where would you place yourself?” Respondents indicated their answer using a 10-point Likert scale (1 being more American and 10 being more Brazilian/Latino/Haitian). Mothers were also asked to compare their dietary intake and physical activity behaviors between their home country and the U.S.; respondents indicated their answer using a 10-point Likert scale (1 being exactly the same and 10 being completely different) [36]. Given that these two questions (comparison of dietary intake and physical activity vs. home country) were highly collinear, we summed the responses (resulting in a range of 1–20) and used it as one variable in the final model.

#### Statistical analysis

According to the scoring procedure for the CFSQ [19], demandingness is calculated as the total mean score across all items, while responsiveness is the ratio of the child-centered items over the total score. Dichotomization at the median within the whole sample was then used to categorize participants into high and low categories on the two dimensions resulting in a typology of the different feeding styles. The median for demandingness in this population was 3.05 and for responsiveness it was 1.11. These medians are similar to those used in other samples which range from 2.6-2.8 for demandingness and 1.14-1.16 for responsiveness [19,25,26,37]. Mean scores for each dimension by feeding styles are as follows: high demanding/high responsive (demandingness: 3.6 ± 0.3, responsiveness: 1.2 ± 0.09); high demanding/low responsive (demandingness: 3.7 ± 0.4, responsiveness: 1.0 ± 0.08); low demanding/high responsive (demandingness: 2.4 ± 0.5, responsiveness:1.3 ± 0.1);low demanding/low responsive (demandingness: 2.5 ± 0.5, responsiveness:1.0 ± 0.08.). Descriptive statistics explored the frequencies, means and standard deviations of feeding styles overall and by immigrant group, acculturation, stress, depression and other covariates (age, education, number of children in the household and marital status). Multiple linear regression models were developed to examine the association between maternal feeding style and child BMI z-score. Based on previous research, we selected the high responsive/high demanding feeding style as the referent category [19,26]. Interactions between ethnicity and feeding styles were also tested. The following variables were adjusted for: child gender and age, maternal age, ethnicity, education, and maternal BMI. Other variables evaluated for inclusion included acculturation (years in country, food and physical activity acculturation), maternal stress, and maternal depression. The final models included participants with complete data (n=236).

## Results

Overall, the average age of the children was 6.2 ± 2.7 years, and 58% of them were male. The mothers’ average age was 36.0 ± 6.4 years, and their average time in the U.S. was 6.0 ± 3.3 years; 31% have less than a high school degree; 36% are Brazilian, 34% are Haitian, and 30% are Latino; and 23% had ≥3 children in the household (Table 1). Seventy-two percent of mothers were overweight and obese while 43% of the children were overweight and obese. Fifteen percent of mothers were categorized as being high demanding/high responsive (e.g., reasoning, complimenting with clear expectations around food consumption); 32% were high demanding/low responsive (e.g., using rewards, punishments without making exceptions or adjustments based on the child’s needs); 34% were low demanding/high responsive (e.g., being warm and accepting but making few demands on the child); and 18% were low demanding/low responsive (e.g., allows her child to do whatever he/she wants).

**Table 1:** Characteristics of the Live Well Study Population According to Feeding Style

	**Total Population**		**High Demanding/High Responsive**		**High Demanding/Low Responsive**		**Low Demanding/High Responsive**		**Low Demanding/Low Responsive**	
	**(n = 383)**		**(n = 59)**		**(n = 123)**		**(n = 129)**		**(n = 68)**	
	**N**	**%**	**N**	**%**	**N**	**%**	**N**	**%**	**N**	**%**
**Socio-demographic Variables**										
**Child Characteristics**										
Male	220	57.6	40	18.4	71	32.7	69	31.8	37	17.1
Age (mean, SD)	6.0	2.7	6.0	2.6	5.7	2.6	6.4	3.0	6.5	2.7
BMIz-score (mean, SD)*	0.9	1.2	0.7^b^	1.2	0.7^b^	1.2	1.2^a^	1.1	1.0 ^a .b^	1.2
Overweight/Obese	164	42.7	24	14.6	46	28.1	67	40.9	27	16.5
**Maternal Characteristics**										
Age (mean, SD)	36.0	6.4	37.0	7.3	35.0	5.9	36.6	6.4	35.5	6.5
Education										
Less than high school	119	31.5	19	16.1	37	31.4	39	33.1	23	19.5
High school, trade/technical school	170	45.0	28	16.6	60	35.5	55	32.5	26	15.4
Some college/college graduate/graduate	89	23.5	12	13.5	25	28.1	34	38.2	18	20.2
Ethnic group^**^										
Brazilian	138	36.0	12	8.8	46	33.6	47	34.3	32	23.4
Latino	114	29.8	19	17.0	23	20.5	55	49.1	21	16.2
Haitian	131	34.2	28	21.5	54	41.5	27	20.8	15	13.4
Marital Status										
Never married	115	30.8	20	17.5	37	32.5	43	37.7	14	12.3
Married	205	55.0	32	15.7	69	33.8	67	32.8	36	17.7
Separated/Divorced/Widowed	53	14.2	5	9.4	14	26.4	18	34.0	16	30.2
Number of children in the household										
1	124	34.2	14	11.4	37	30.1	46	37.4	26	21.1
2	156	43.0	30	19.4	48	31.0	49	31.6	28	18.1
≥3	83	22.9	12	14.5	33	39.8	28	33.7	10	12.1
**Acculturation Variables**										
Years of residence in US (Mean, SD)	6.0	3.3	5.6	3.3	5.8	3.2	6.3	3.0	6.0	3.4
When you think about your daily life now, where would you place yourself on the scale (1 being more American and 10 being more Brazilian/Latino/Haitian)										
(Mean, SD)	7.9	2.2	7.7	2.4	8.1	2.2	7.9	2.3	7.6	2.1
How would you compare the food/physical activity you normally eat/do in the US vs. what you ate/did in your home country?										
(Mean score, SD)	14.1	4.6	14.9	4.2	14.3	4.7	13.7	4.4	14.1	5.1
BMI (kg/m^2^) (Mean, SD)	28.7	5.8	28.6	6.2	27.6	5.1	29.3	5.8	29.5	6.4
**Behavioral Variables**										
Perceived Stess Scale (mean, SD)*	16.9	5.9	17.5	5.5	17.7^a^	5.5	15.6^b^	6.0	17.5	6.2
Depression scale (mean, SD)	15.0	9.9	15.9	10.6	15.9	9.5	13.2	10.0	15.7	9.5

Sample sizes vary slightly due to missing data.

*p < 0.01,**p < 0.0001.

P-values generated from Chi-square tests and Fisher's exact for cells that have expected counts < 5.

^a,b^ superscripts signifies significant difference (P < 0.05).

In bivariate analyses, child BMI z-score, perceived stress and ethnic group differed across feeding styles (Table 1). Post hoc analysis revealed that mothers who expressed a low demanding/high responsive feeding style had children with a higher BMI z-score compared to the other feeding style groups (1.2 vs. 0.7 for high demanding/high responsive; 0.7 for high demanding/low responsive, p = 0.002). Post hoc analysis also showed that women with a high demanding/low responsive feeding style had a higher perceived stress score as compared to the low demanding/high responsive feeding style (17.7 vs. 15.6, p = 0.02). A larger percentage of Haitian women belong to the high demanding/low responsive feeding style compared to Brazilians and Latinas (42% vs. 34% and 21% respectively), while a higher percentage of Latina women belong to the low demanding/high responsive style as compared to Brazilian and Haitian (49% vs. 34% and 21%, respectively).

In multiple linear regression, a low demanding/high responsive feeding style was positively associated (ß = 0.56) with a higher BMI z-score (p = 0.01) after controlling for known covariates. This model accounted for 26% of the variation in a child’s BMI z-score (Table 2). No statistically significant interactions were observed between ethnic group and feeding style.

**Table 2:** Beta Coefficients and Standard Errors from a Linear Regression Model for BMIz Scores

Feeding Styles	**BMI-Z**					
	**Unadjusted Model**			Adjusted Model*		
	**B**	**SE**	**p value**	**B**	**SE**	**p value**
High Demanding/High Responsive	REF			REF		
High Demanding/Low Responsive	−0.06	0.19	0.76	−0.20	0.21	0.35
Low Demanding/High Responsive	0.50	0.18	0.01	0.56	0.21	0.01
Low Demanding/Low Responsive	0.23	0.21	0.26	0.14	0.25	0.57

*Models adjusted for child gender and age, maternal age, ethnicity, education, BMI, perceived stress, depression, years in the United States.

## Discussion

We found that among this diverse group of mothers, the majority are categorized into a high demanding/low responsive or low demanding/high responsive feeding style. Feeding styles differed by child weight status, ethnicity and perceived stress. In multivariate analysis, we found that a low demanding/high responsive feeding style was significantly and positively associated with child weight status.

Our finding that a large number of mothers were categorized as high demanding/low responsive and low demanding/high responsive adds to the growing literature of what others have found in studies with diverse, low-income parents. Ventura and colleagues found that among a small sample of diverse parents, only 15% were categorized into the high demanding/high responsive style [38] whereby parents may negotiate or praise their child. This finding is consistent with findings in our population, where only 17% of mothers were categorized into high demanding/high responsive style. Similarly the majority of African American/Hispanic mothers reported from a study of Head Start parents were classified into the same two feeding styles: high demanding/low responsive and low demanding/high responsive [19]. Olvera and colleagues also found a small percentage of Mexican American mothers who were categorized as high demanding/high responsive in their general parenting style [39]. Because parents’ attitudes toward child-rearing are influenced by cultural norms and other contextual factors, we expected to identify ethnic differences across feeding styles. These differences have also been observed in the general parenting style literature [38,40,41].

Although there has been some investigation into how maternal mental health, including stress and depression, can interfere with responsive parenting [42-45], few studies have explored associations between maternal mental health and feeding styles. In our bivariate analysis, we found that mothers with higher stress scores were also more likely to express high demanding/low responsive feeding style. Hurley and colleagues conducted a study of 702 diverse mother-infant dyads and found that maternal stress and depression was significantly associated with both a forceful and an uninvolved feeding style in mothers of African-American children [46]. Although our finding that a higher demanding/less responsive style appears to be operating in the same direction, the population in this study was different than ours (African American infants), and the feeding styles were assessed with a different measure, thus limiting comparisons. Future studies should further explore how mental health may influence feeding dynamics in diverse populations of parents.

Our finding that a low demanding/high responsive feeding style is predictive of higher child weight is consistent with our hypothesis and is supported by prior literature with diverse populations. Hennessy and colleagues found this style was significantly associated with both higher child weight status and consumption of low-nutrient dense foods among a rural population of mostly African-American and Hispanic mothers [26,47]. Similarly, Hughes and colleagues found that among 718 Head Start parents, predominantly African American and Hispanic, a low demanding/high responsive feeding style was significantly associated with a high child body mass index controlling for parent affect, child temperament and other covariates [19]. In this sample, a low demanding/high responsive style was also associated with low consumption of fruits and vegetables [23]. Together, these results suggest that that parents with a low demanding/high responsive style try to control the emotional climate of specific feeding occasions by being nice, supportive, and non-directive, which may result in a child overeating and gaining unnecessary weight.

In the context of Live Well, it is possible that these immigrant mothers may be transitioning from under resourced countries where they often had to say “no” to their child’s requests and that moving into an “obesogenic” food environment where high energy dense food is more readily available and less costly they feel this is an area where they can say “yes” to their child more often. This would help explain how a low demanding/high responsive feeding style would be associated with a higher child weight status in our study population. Moreover, it is likely that immigrant mothers have other life stressors that take a higher priority in their lives than feeding situations, and that this, in turn may influence the feeding environment [8]. Given this high level of stress associated with the acculturative process, mothers may find it easier to say “yes” to food requests. In our study, we did not identify associations between acculturation (time of residence in the U.S., and food and physical activity changes) and feeding styles; however, these proxies do not capture specific aspects of the transition from one country to another, such as attitudes towards certain feeding environments (e.g., fast food restaurants) or types of foods (e.g., chips or soda), which may have influenced feeding interactions. Future research is needed to help understand the larger socio-cultural context, its effects on the mental health of our participants and its influence on feeding interactions among immigrant families. It is also important to note that our study took place during a time of strong anti-immigrant feelings in general and actions against undocumented immigrants in particular. Simultaneously, social upheaval in the country of origin and the January 2010 Haitian earthquake added considerable situational stress for that community.

Our findings should be interpreted within the context of the study limitations. Although our sample represents a diverse group of mother–child dyads, generalizability may still be limited given the focus on families from Brazil, Haiti and Latin America (predominately Central and South America). As a cross-sectional study, one cannot discern the direction of influence, nor look at change over time. For example, we do not know if these children were overweight or obese before coming to the US and whether feeding styles changed based on their location. Further, mothers are not parenting in isolation, but in response to several factors including child traits [48]. Thus, the parent–child relationship is bi-directional and additional studies are needed to understand causal pathways between parenting behaviors (from both mothers and fathers) and child weight status that also include measures of specific child behaviors (e.g., food intake). Although our measures captured some aspects of acculturation, we did not gather specific information on how the acculturative process may influence the feeding dynamics or other aspects of socio-cultural norms.

## Conclusions

In conclusion, we found that a low demanding/high responsive feeding style is significantly and positively associated with child weight status among a group of recent immigrant mothers, even after adjusting for ethnicity, acculturation and stress. Our findings add to the growing literature suggesting that parents need to be aware of potential unintended consequences of their feeding styles. Given that the risk of obesity increases with duration of residence in the U.S., exploring the role of possible risk factors, such as feeding styles, is critical. Future prospective studies with other immigrant populations are needed to confirm our findings, and to identify socio-cultural factors that influence the feeding dynamic, in order to inform culturally-appropriate prevention and treatment programs.

## Competing interest

The authors declare that they have no competing interests.

## Authors’ contributions

All the authors contributed to the various stages of this study. AT contributed to the study design, performed all of the statistical analysis, and drafted the manuscript. EH contributed to the study design and helped draft the manuscript. AP, AM, DG and RH participated in the design of the study and revised manuscript. SS, RB, CLK and HG collected data and revised manuscript. SH participated in the design of the study and revised manuscript. CDE conceived of the initial idea of the study, contributed to design of the study, revised the manuscript and contributed especially to the intellectual content. All the authors read and commented on the drafts and approved of the final version for submission.
